# Infliximab-Associated Psoriasiform Dermatitis: Case Report and Review of a Seemingly Paradoxical Inflammatory Response

**DOI:** 10.7759/cureus.773

**Published:** 2016-09-09

**Authors:** Tiffany Y Loh, Philip R Cohen

**Affiliations:** 1 School of Medicine, University of California, San Diego; 2 Department of Dermatology, University of California, San Diego

**Keywords:** bowel, crohn’s, dermatitis, inflammatory, infliximab, interferon, psoriasiform, spongiotic, ulcerative colitis, tumor necrosis factor

## Abstract

Background: Tumor necrosis factor-α (TNF-α) inhibitors, such as infliximab, adalimumab, and certolizumab pegol are effective agents in the treatment of inflammatory bowel disease. Some individuals undergoing anti-TNF-α therapy for Crohn’s disease or ulcerative colitis develop psoriasiform lesions. This is a paradoxical finding, as classical psoriasis is known to respond to these agents.

Purpose: The clinical features of anti-TNF-α-induced psoriatic dermatitis are described.

Method: A 60-year-old man with Crohn’s disease treated with infliximab, who developed anti-TNF-α-induced psoriasiform dermatitis, is described.

Results: The man developed erythematous skin lesions in the bilateral axillae two years after beginning infliximab treatment for Crohn’s disease. Biopsy revealed psoriasiform dermatitis, consistent with a diagnosis of anti-TNF-α-induced psoriasiform dermatitis. He was treated with clobetasol 0.05% ointment twice daily for two weeks and had significant improvement. Subsequently, he used the corticosteroid ointment two days per week and calcipotriene 0.005% ointment twice daily for five days per week to achieve and maintain clear skin.

Conclusions: Anti-TNF-α-induced psoriasiform dermatitis is an infrequent complication of infliximab therapy. However, the condition may require discontinuation of the anti-TNF-α agent. Anti-TNF-α-induced psoriasiform dermatitis should be considered in the differential diagnosis when evaluating a new erythematous skin condition in an individual with a history of inflammatory bowel disease who is being treated with a TNF-α inhibitor.

## Introduction

Tumor necrosis factor-α (TNF-α) inhibitors are used to treat both inflammatory bowel disease and psoriasis [1]. However, paradoxically, the development of psoriatic lesions has been observed in some individuals after the initiation of TNF-α inhibitor therapy for treatment of Crohn’s disease and ulcerative colitis [2]. Specifically, infliximab, adalimumab, and certolizumab pegol have been associated with psoriasiform dermatitis, and several studies have postulated that the development of this dermatosis may represent a drug reaction [2]. Herein, we present a case of infliximab-induced psoriasiform dermatitis and review the features of TNF-α inhibitor-induced psoriasiform dermatitis. 

## Case presentation

A 60-year-old man, with an eight-year history of Crohn’s disease, had been receiving infliximab and azathioprine for two years and remained in clinical remission. He developed scaly red plaques in the bilateral axillae that were initially treated with ciclopirox amine 0.77% cream twice daily. There was minimal improvement during the next six months. 

**Figure 1:**
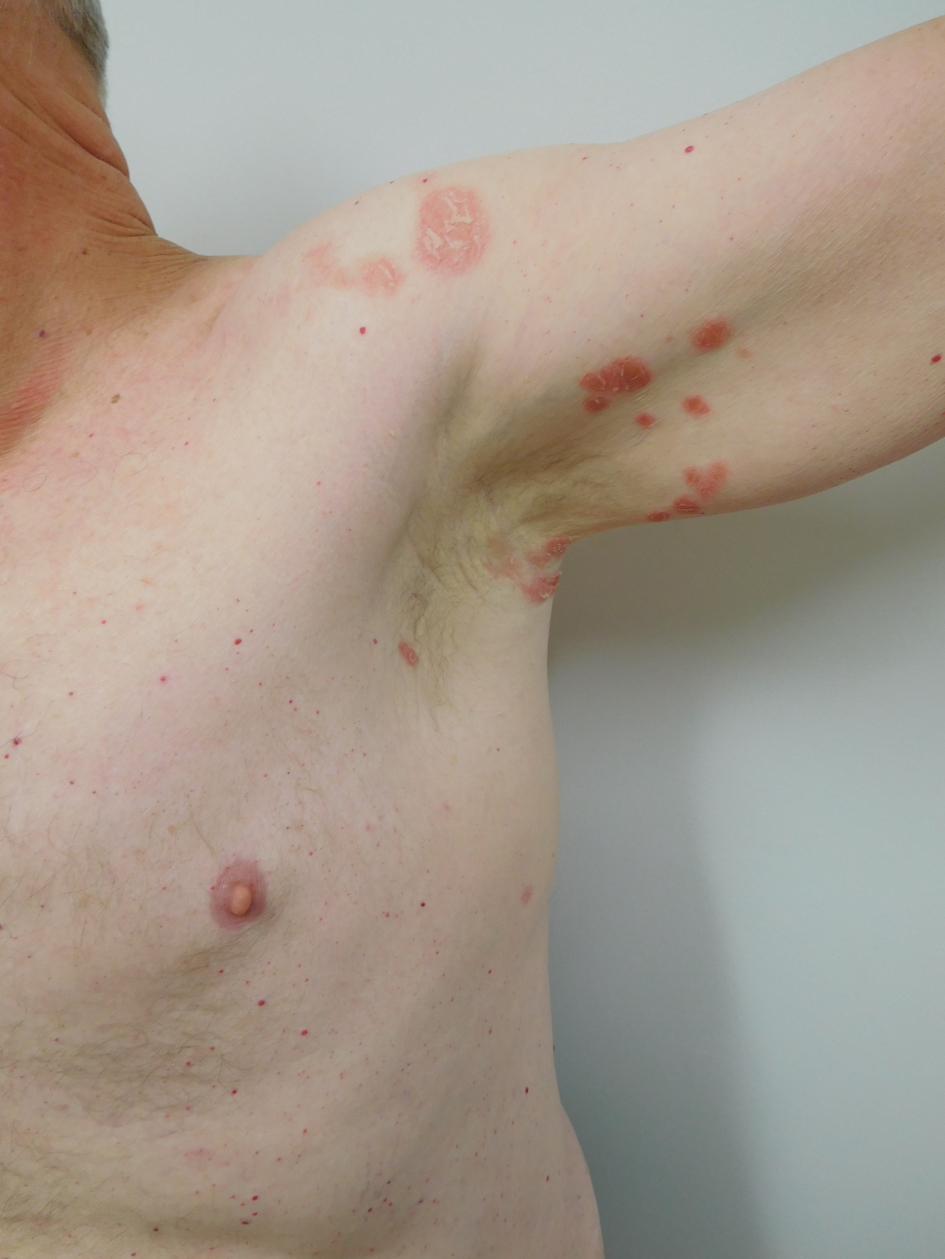
Distant View of Psoriasiform Dermatitis of Left Axilla Distant view of the scaly red plaques in the left axilla that appeared two years after initiation of infliximab therapy in a man with Crohn's disease.

**Figure 2:**
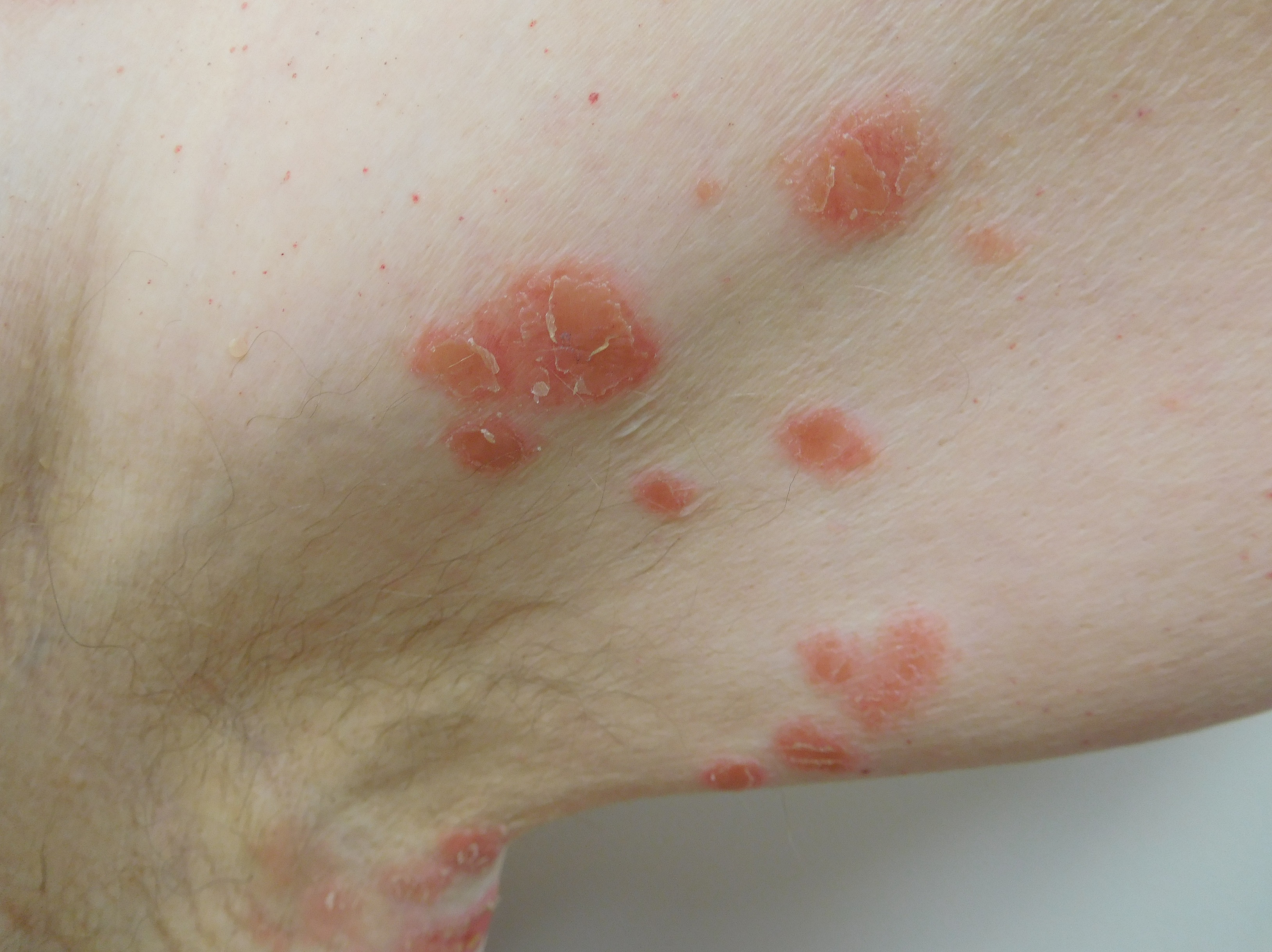
Closer View of Psoriasiform Dermatitis of the Left Axilla Closer view of the scaly red plaques in the left axilla that appeared two years after initiation of infliximab therapy in a man with Crohn's disease.

**Figure 3:**
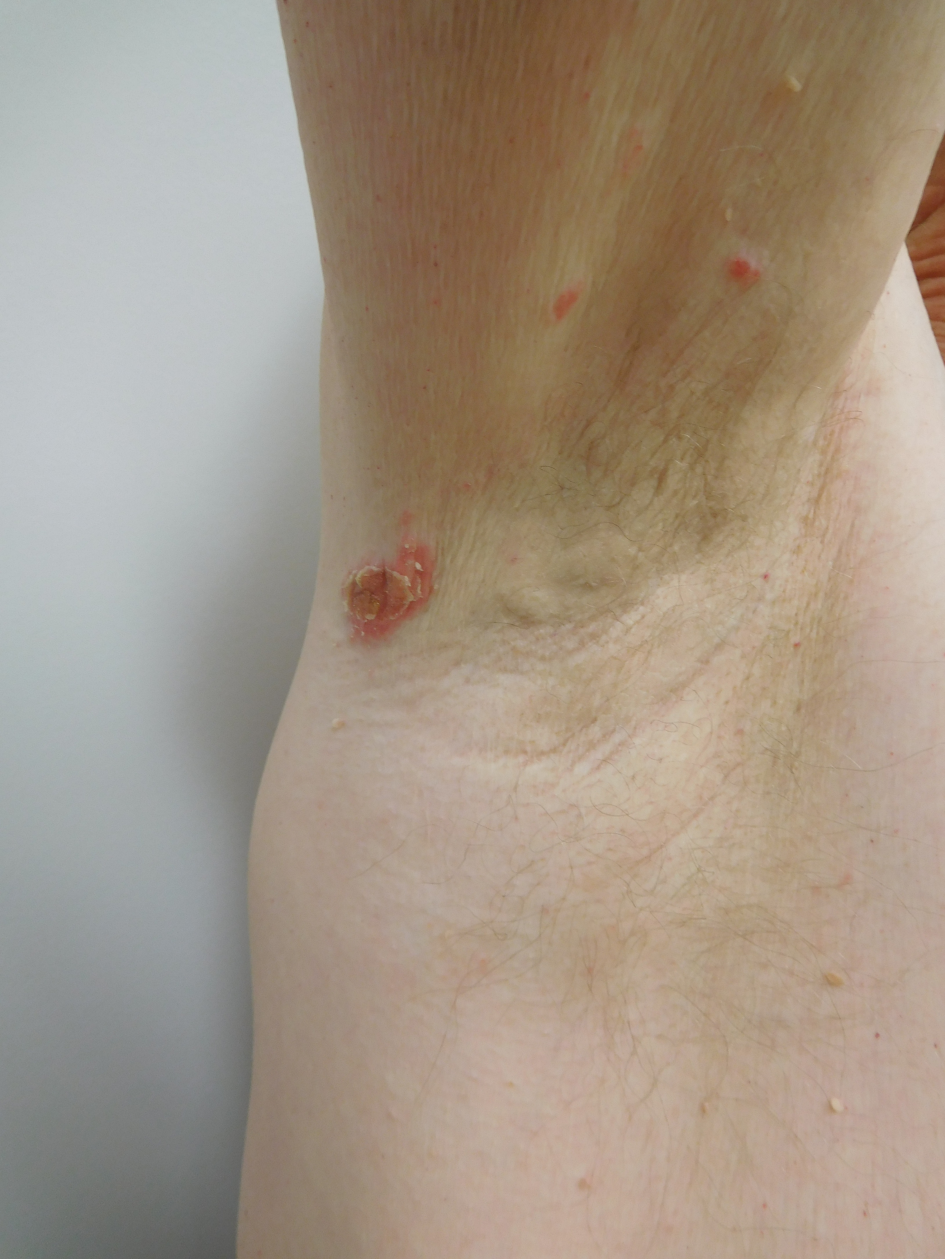
Distant View of Psoriasiform Dermatitis of the Right Axilla Distant view of the scaly red plaques in the right axilla that appeared two years after initiation of infliximab therapy in a man with Crohn's disease.

**Figure 4:**
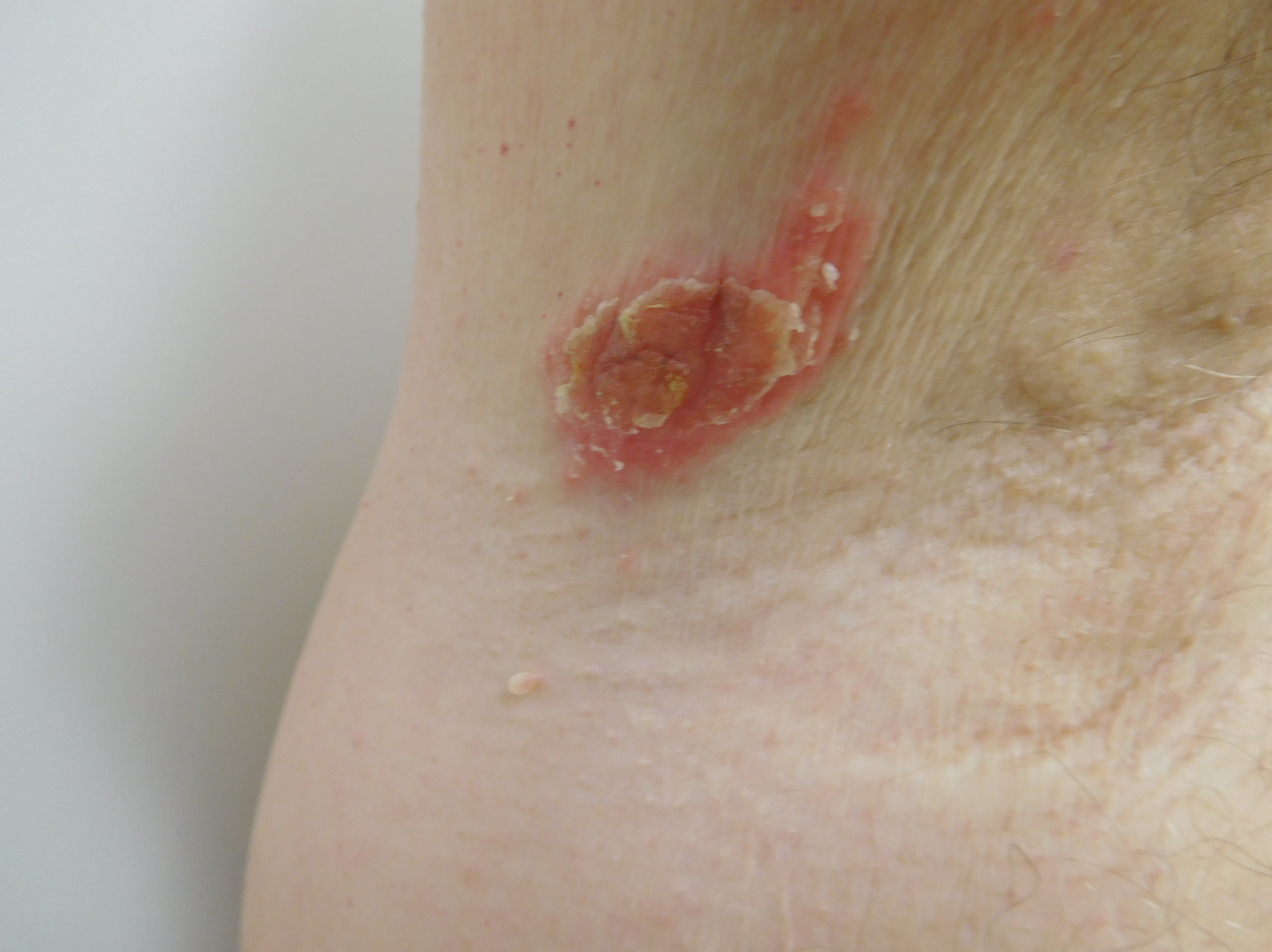
Psoriasiform Dermatitis Closer view of the scaly red plaques in the right axilla that appeared two years after initiation of infliximab therapy in a man with Crohn's disease.

When he subsequently presented for evaluation, physical examination revealed eight scaly red plaques in the left axilla and one plaque in the right axilla, all measuring between 1-3 cm in the greatest diameter. The patient consented to have his photos used in this case report (Figures 1-4).

**Figure 5:**
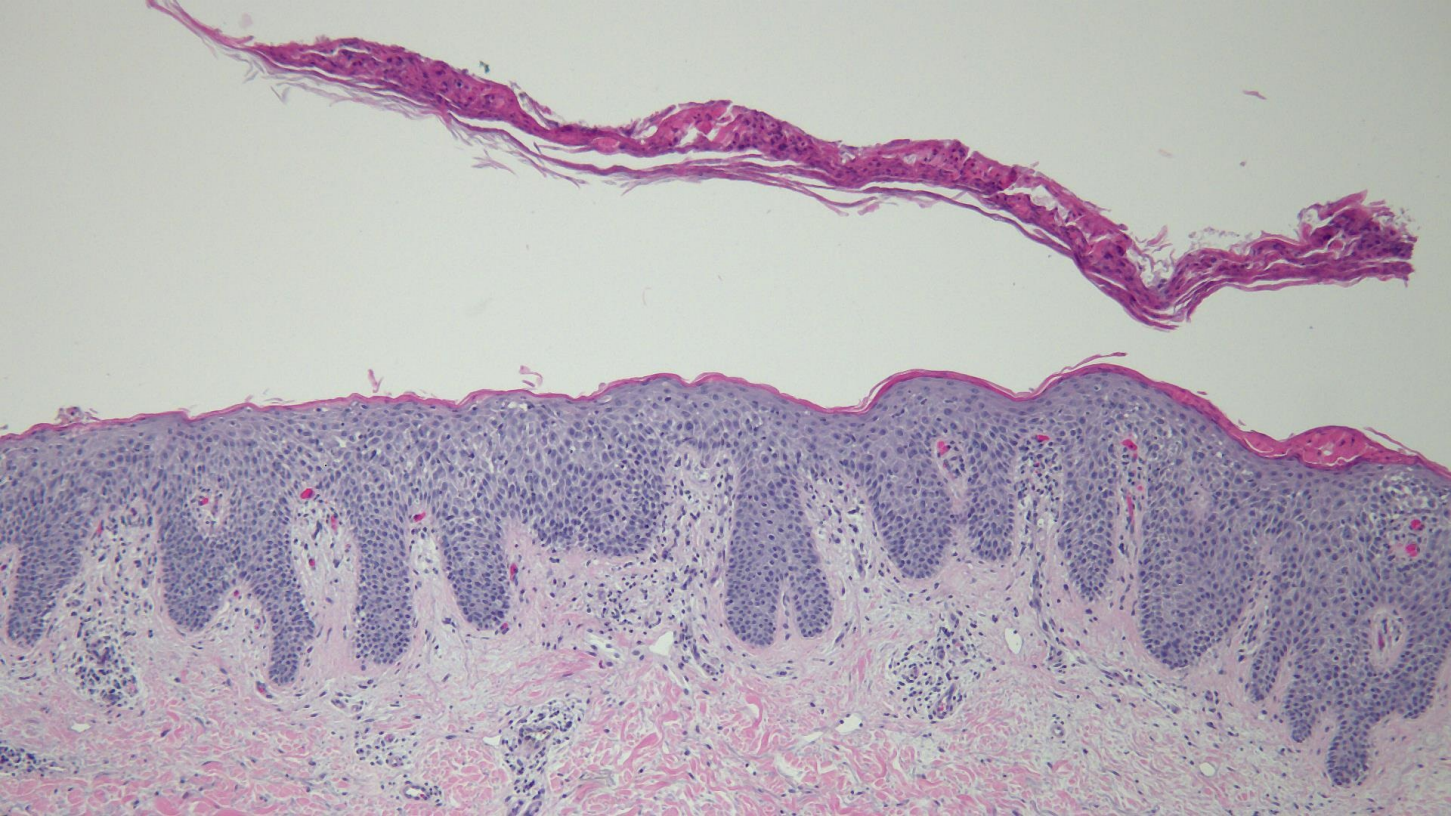
Low Magnification View of Biopsy Specimen from the Left Axilla The stratum corneum shows parakeratosis with collections of serum and neutrophils. There are acanthosis and elongation of the rete ridges into the upper dermis (hematoxylin and eosin; x4).

**Figure 6:**
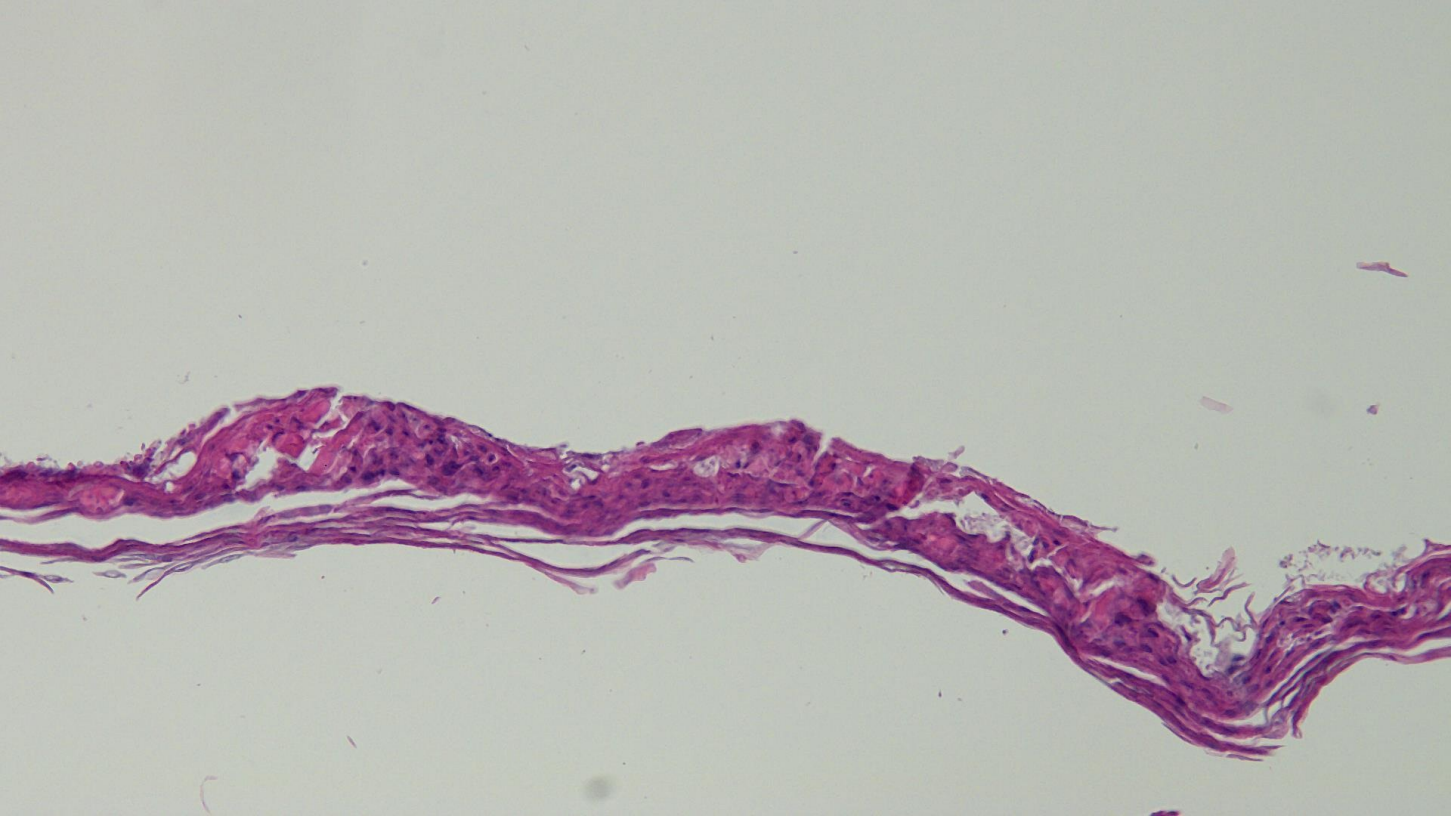
Intermediate Magnification View of Stratum Corneum from the Biopsy Specimen from the Left Axilla The stratum corneum shows parakeratosis with collections of serum and neutrophils (hematoxylin and eosin; x10).

**Figure 7:**
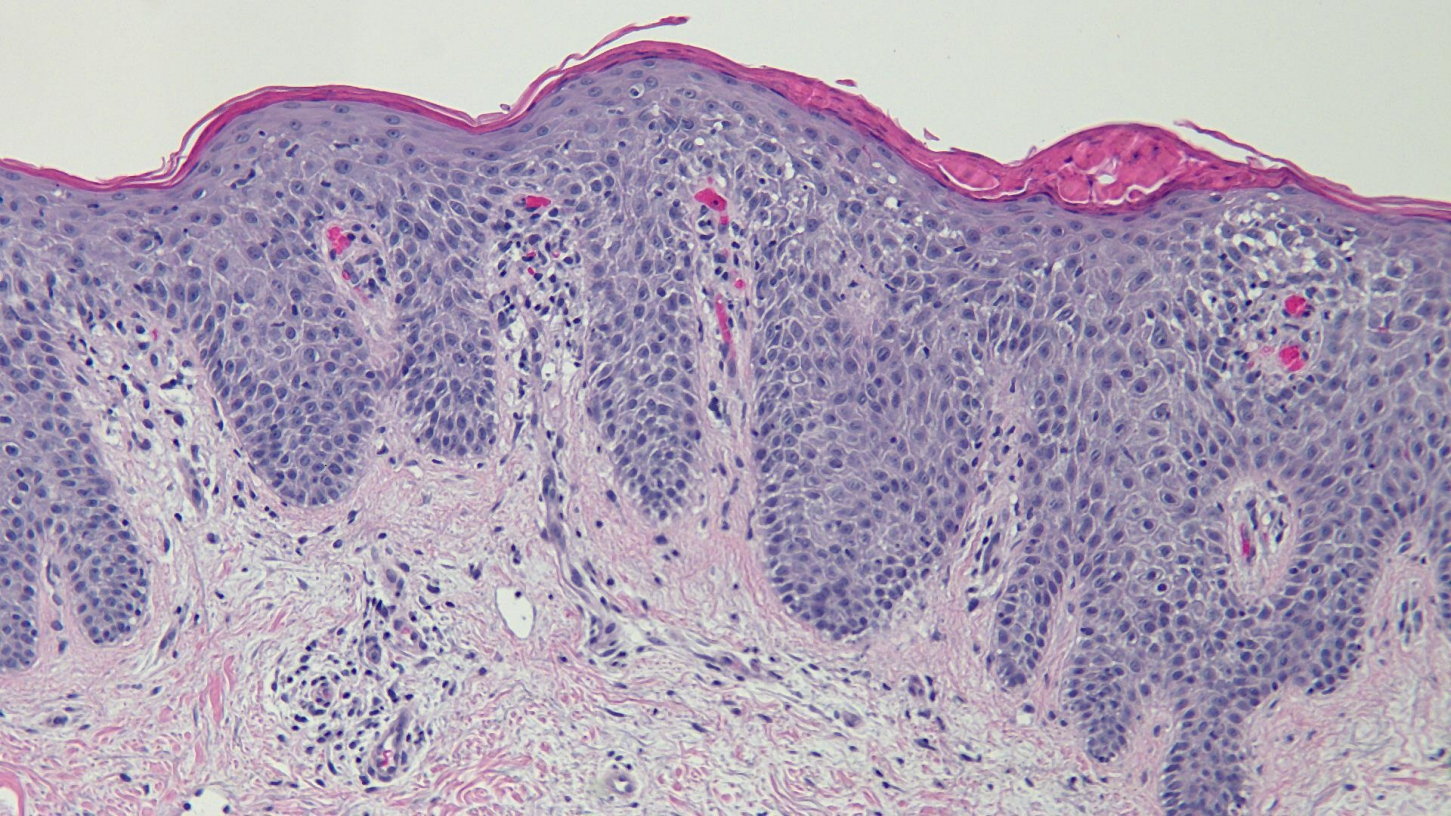
Intermediate Magnification View of the Biopsy Specimen from the Left Axilla The stratum corneum shows parakeratosis with collections of serum and neutrophils. There are acanthosis and elongation of the rete ridges into the upper dermis. The granular layer is diminished, and there is spongiosis with neutrophils in the epidermis (hematoxylin and eosin; x10).

**Figure 8:**
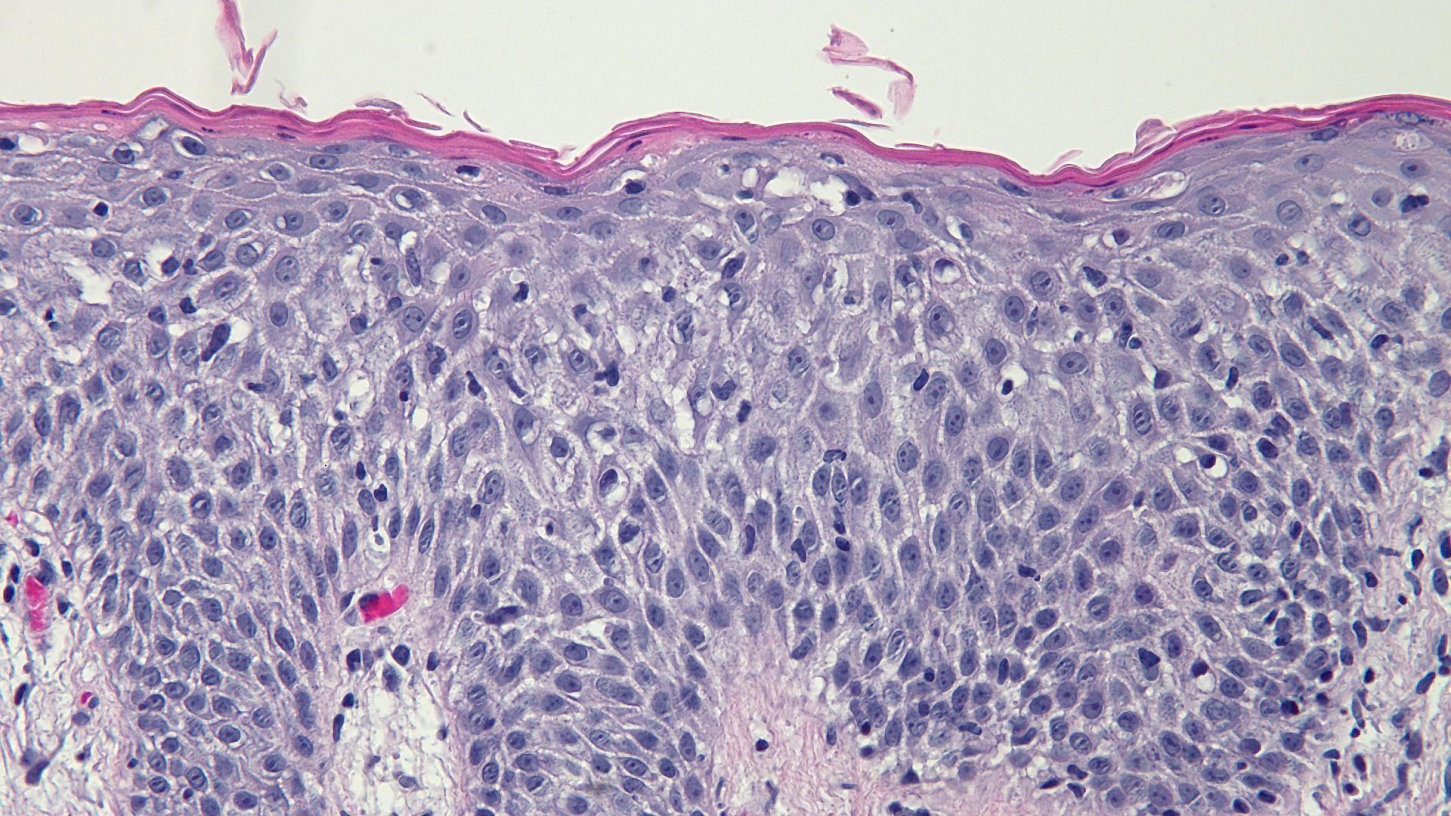
High Magnification View of the Biopsy Specimen from the Left Axilla There are acanthosis and elongation of the rete ridges into the upper dermis. The granular layer is diminished, and there is spongiosis with neutrophils in the epidermis (hematoxylin and eosin; x20).

Biopsy of two lesions showed similar findings (Figures 5-8). The stratum corneum had parakeratosis with collections of serum and neutrophils. The epidermis was acanthotic and there was an elongation of the rete ridges into the upper dermis; in addition, the granular layer was diminished, and there was spongiosis.

Correlation of the clinical presentation, cutaneous morphology, and pathology changes established a diagnosis of TNF-α inhibitor-associated psoriasiform dermatitis. Initial treatment was clobetasol 0.05% ointment twice daily. Two-week follow-up revealed significant improvement of the skin lesions. Subsequently, he used the corticosteroid ointment two days per week and calcipotriene 0.005% ointment twice daily for five days per week to achieve and maintain clear skin.

## Discussion

The occurrence of psoriasis-like lesions associated with anti-TNF-α therapy is infrequent, with an incidence rate of 1.04 to 3.0 per 1,000 person-years [2]. The development of this dermatosis may adversely affect the treatment of inflammatory bowel disease, including the possibility of discontinuing anti-TNF-α therapy. In addition, the uncommon occurrence and variable clinical presentation of TNF-α inhibitor-associated psoriasiform dermatitis may make for a challenging diagnosis.

Although several studies have attempted to elucidate the mechanism of anti-TNF-α-induced psoriasiform dermatitis, the pathogenesis of this condition remains unclear. Recent theories postulate that increased interferon-α levels, which are associated with reduced TNF-α, may contribute to the development of psoriatic plaques [3-4]. Systemic and topical interferon-α treatment has been observed to exacerbate psoriasis, which supports the theory that anti-TNF-α agents may induce development of psoriatic skin lesions [5-6]. Studies have shown that inhibition of TNF-α leads to activation of autoreactive T cells and increased production of interferon-α by dendritic cells, which has been linked to the development of psoriasis [4].

Decreased TNF-α levels appear to be associated with increased levels of pro-inflammatory cytokines, such as interleukin-12, interleukin-17, and interleukin-23, which may also contribute to the inflammatory reaction [3, 7]. 

Although the lesions of classical psoriasis and anti-TNF-α-induced psoriasiform dermatitis appear similar, their management may differ. Classical psoriasis can be treated with a variety of topical (corticosteroids and vitamin D analogs) and/or systemic (antimetabolites, immunomodulators, TNF inhibitors, and interleukin inhibitors) agents. In contrast, anti-TNF-α-induced psoriasiform dermatitis may only be resolved with the discontinuation of the anti-TNF-α agent or switching to another TNF-α inhibitor [2, 8].

As demonstrated by our patient’s case, anti-TNF-α-induced psoriasiform dermatitis may be responsive to high-potency topical corticosteroids. Consistent with the observations, several studies also report the use of topical corticosteroids as the initial treatment for anti-TNF-α-induced psoriasiform dermatitis, with response rates ranging from 40-47% [2, 7]. In those who fail to respond to topical corticosteroids, further treatment options include switching to a second anti-TNF-α agent or stopping anti-TNF-α therapy altogether. However, recurrence of the rash on the second anti-TNF-α agent is common (90% as reported by Rahier, et al. [6]), and ultimately, up to 52% of these individuals stop anti-TNF-α therapy due to the psoriasiform dermatitis [2, 6].

Although the occurrence of psoriatic dermatitis in association with anti-TNF-α therapy for inflammatory bowel disease is a rare occurrence, this complication involves considerable morbidity. Several studies have documented the difficulty of obtaining remission from the skin lesions [2]. Cullen, et al. found that although 41% of 148 individuals who developed a psoriasiform dermatitis responded to topical therapy and were able to continue the medication, 43% ultimately required withdrawal of the anti-TNF-α agent due to dermatologic concerns [2]. Twenty-seven individuals attempted an alternate TNF-α inhibitor, but 14 (52%) of them experienced persistence or recurrence of the dermatosis. 

Additionally, Fréling, et al. found in a study of 538 inflammatory bowel disease patients that 59 individuals (10.1%) developed psoriasiform lesions (median follow-up period of 38.2 months) [9]. For individuals who switched to a second anti-TNF-α agent, 57% experienced recurrence of the lesions. Some studies have proposed that the recurrence of disease despite a change in anti-TNF-α therapy suggests that the individuals involved in these cases may have a predisposition to developing psoriasis and that those who experience resolution may have had a true drug-induced reaction [2]. However, the mechanism underlying the development of these lesions is still unclear, and more studies are warranted.

Various risk factors appear to be associated with the development of psoriasiform skin lesions in patients receiving TNF-α inhibitors. Women appear to be more commonly affected than men [2]. Also, individuals with Crohn’s disease appear to be at higher risk of developing this complication in comparison to patients with ulcerative colitis [2-3, 6, 9].

In addition, infliximab has been implicated in the majority of cases involving anti-TNF-α-induced psoriasiform dermatitis [2]. However, this association may be due to the fact that infliximab is an older drug that is more commonly used than the other TNF-α inhibitors [2]. The relationship between specific anti-TNF-α agents and the development of psoriasiform skin lesions may be elucidated with additional investigation of these patients.

Several characteristics of anti-TNF-α-associated psoriasiform skin lesions may distinguish them from classical psoriasis. Contrary to psoriasis, in which lesions are typically found on the extensor surfaces, such as the knees and elbows, there is a high prevalence of palmoplantar and scalp involvement in the patients with anti-TNF-α-induced psoriasiform dermatitis [2]. In addition, palmoplantar pustulosis, which is less common in psoriasis, has been found to be more frequent in patients with anti-TNF-α therapy-associated skin lesions [2]. These observations suggest that although the lesions of classical psoriasis and anti-TNF-α-induced psoriasis may clinically appear similar, the underlying mechanism of their development may differ, which may have implications for treatment interventions.

Interestingly, most patients with anti-TNF-α-induced psoriasiform skin lesions do not have a history of psoriasis [2, 6]. In patients who do have a personal history of psoriasis, the drug-induced lesions appear in previously unaffected sites and commonly have an atypical appearance [2].

The onset of psoriasiform skin lesions after initiation of anti-TNF-α therapy is highly variable. In a 2009 review of 127 cases, psoriasiform dermatitis was found to occur after an average of 10.5 months after the initiation of the biologic agents; however, some cases have been reported to occur merely days after initiation of therapy, while others do not occur until up to four years after beginning treatment [10]. Our patient presented with psoriasiform dermatitis approximately two years after beginning infliximab therapy.

The variable onset of TNF-α inhibitor-induced psoriasiform dermatitis suggests that, in addition to genetic factors that may predispose certain individuals to developing this condition, environmental influences may also be contributing [2]. However, no specific environmental triggers have been identified.

## Conclusions

The development of anti-TNF-α-induced psoriasiform dermatitis has been associated with infliximab, adalimumab, and certolizumab pegol. However, TNF-α-inhibitor-induced psoriasiform dermatitis is uncommon and the skin lesions may be easily mistaken for either classical psoriasis or other dermatoses. Special attention to the characteristics and distribution of the lesions is necessary to make an accurate diagnosis. Risk factors for the development of anti-TNF-α-induced psoriasiform dermatitis include female gender, no personal history of psoriasis, and the presence of Crohn’s disease. The onset of the skin lesions is unpredictable, ranging from a few days to several years, and the presentation may be highly variable. Nevertheless, certain characteristics may indicate that the consideration of an anti-TNF-α-induced psoriasiform dermatitis is appropriate. These features include involvement of the palms, soles, and scalp regions, the presence of palmoplantar pustulosis, occurrence in individuals without a previous history of psoriasis, and the dermatosis occurring in previously uninvolved areas in patients who do have psoriasis. Treatment for classical psoriasis and anti-TNF-α-induced psoriasiform dermatitis both include topical corticosteroids. Delay in diagnosis and appropriate treatment may lead to significant morbidity in patients with TNF-α-inhibitor-induced psoriasiform dermatitis. Therefore, a high index of suspicion for anti-TNF-α-induced psoriasiform dermatitis is needed when considering the differential diagnosis for erythematous papules and plaques in a patient with inflammatory bowel disease who is receiving anti-TNF-α therapy.
